# Pharmacokinetic-Pharmacodynamic Assessment of the Hepatic and Bone Marrow Toxicities of the New Trypanoside Fexinidazole

**DOI:** 10.1128/AAC.02515-18

**Published:** 2019-03-27

**Authors:** James A. Watson, Nathalie Strub-Wourgraft, Antoine Tarral, Isabela Ribeiro, Joel Tarning, Nicholas J. White

**Affiliations:** a Mahidol-Oxford Tropical Medicine Research Unit, Faculty of Tropical Medicine, Mahidol University, Bangkok, Thailand; b Centre for Tropical Medicine and Global Health, Nuffield Department of Medicine, University of Oxford, Oxford, United Kingdom; c Drugs for Neglected Diseases *initiative* (DND*i*), Geneva, Switzerland

**Keywords:** Chagas disease, Gambian sleeping sickness, fexinidazole, liver toxicity, neutropenia, nitroimidazole

## Abstract

Fexinidazole is a novel oral treatment for human African trypanosomiasis caused by Trypanosoma brucei gambiense (*g*-HAT). Fexinidazole also has activity against T. cruzi, the causative agent of Chagas disease. During the course of a dose ranging assessment in patients with chronic indeterminate Chagas disease, delayed neutropenia and significant increases in hepatic transaminases were observed and clinical investigations were suspended. We retrospectively analyzed all available pharmacokinetic and pharmacodynamic data on fexinidazole in normal healthy volunteers and in patients with Chagas disease and *g*-HAT to assess the determinants of toxicity. A population pharmacokinetic model was fitted to plasma concentrations (*n* = 4,549) of the bioactive fexinidazole sulfone metabolite, accounting for the majority of the bioactive exposure, from three phase 1 studies, two *g*-HAT phase 2/3 field trials, and one Chagas disease phase 2 field trial (*n* = 462 individuals in total). Bayesian exposure-response models were then fitted to hematological and liver-related pharmacodynamic outcomes in Chagas disease patients. Neutropenia, reductions in platelet counts, and elevations in liver transaminases were all found to be exposure dependent and, thus, dose dependent in patients with Chagas disease. Clinically insignificant transient reductions in neutrophil and platelet counts consistent with these exposure-response relationships were observed in patients with *g*-HAT. In contrast, no evidence of hepatotoxicity was observed in patients with *g*-HAT. Fexinidazole treatment results in a dose-dependent liver toxicity and transient bone marrow suppression in Chagas disease patients. Regimens of shorter duration should be evaluated in clinical trials with patients with Chagas disease. The currently recommended regimen for sleeping sickness provides exposures within a satisfactory safety margin for bone marrow suppression and does not cause hepatotoxicity.

## Abstract (precis)

Fexinidazole is a novel oral treatment for human African trypanosomiasis caused by Trypanosoma brucei gambiense (*g*-HAT). Fexinidazole also has activity against T. cruzi, the causative agent of Chagas disease.

## TEXT

The nitroimidazole fexinidazole is a promising new treatment for sleeping sickness (human African trypanosomiasis caused by Trypanosoma brucei gambiense [*g*-HAT]), which has recently received approval by the European Medicines Agency (1). Fexinidazole has the potential to become the first-line therapy for most cases of Gambian sleeping sickness (1–4). It is an oral treatment for both the blood stage and the central nervous system (CNS) stage of the disease. Fexinidazole is metabolized extensively *in vivo* to two biologically active metabolites, a sulfoxide (M1) and a more slowly eliminated sulfone (M2) (5, 6). The sulfone metabolite accounts for the majority of bioactive exposure during the 10 days of fexinidazole treatment currently recommended in patients with *g*-HAT (7). Phase 2 and 3 studies of fexinidazole in patients with *g*-HAT have shown it to be efficacious and well tolerated. This class of drugs also has good *in vitro* and *in vivo* (murine model) activity against other kinetoplastid parasites: T. cruzi (6), T. lewisi (8), and Leishmania donovani (9). Clinical studies have been performed in both Chagas disease and visceral leishmaniasis patients. An extended dose-finding study was conducted in patients with chronic indeterminate Chagas disease (hereafter denoted chronic Chagas disease), in which the maximum duration of treatment was 8 weeks, as it is now for benznidazole, the current treatment of choice. In the course of this study, increases in hepatic transaminases and a delayed and transient fall in neutrophil counts were noted. In response to these findings, clinical studies in chronic Chagas disease patients were halted temporarily and additional hematology and liver function investigations were added to ongoing studies in *g*-HAT patients. As the prospective *g*-HAT studies had not anticipated this toxicity when they started, a retrospective pharmacokinetic-pharmacodynamic assessment was conducted using all the available clinical data to characterize the relationships between drug and metabolite exposures and potential adverse effects and to provide predictions for the future safety of the 10-day fexinidazole regimen developed for the treatment of *g*-HAT.

## RESULTS

### Population pharmacokinetic model of fexinidazole sulfone (M2).

Pharmacokinetic data quantifying the concentrations of the M2 metabolite in 462 individuals over more than 4,500 time points were analyzed jointly. An overall summary of the six data sets used in this analysis is shown in Table 1.

**TABLE 1:** Summary of the available fexinidazole pharmacokinetic data^*a*^

Trial type	Trial name	Country	No. of subjects			Median (range) no. of samples/person	Median (range) total dose (mg/kg)
			Total	Males	Females		
Phase 1	FEX001	France^*b*^	71	71	0	17 (17–20)	1.4 (1–813)
Phase 1	FEX002	France^*b*^	12	12	0	15	45 (41–61)
Phase 1	FEX003	France^*b*^	22	22	0	17 (11–17)	178 (19–229)
*g*-HAT	FEX004	CAR, DRC	203	125	78	6 (3–6)	228 (195–480)
*g*-HAT	FEX006	CAR, DRC	114	64	50	5 (4–5)	336 (247–420)
Chagas	CH-FEX001	Bolivia	40	12	28	3 (1–6)	251 (61–1400)

a These studies relate only to the quantification of the major bioactive metabolite M2. The phase 1 data were used to evaluate the best structural model, and the full data set was used in the pharmacokinetic analyses in order to estimate the drug exposures in fexinidazole-treated chronic indeterminate Chagas disease patients and fexinidazole-treated *g*-HAT patients. CAR, Central African Republic; DRC, Democratic Republic of Congo.

b Phase 1 studies in France were conducted in healthy volunteers of sub-Saharan African origin.

**FIG 1:**
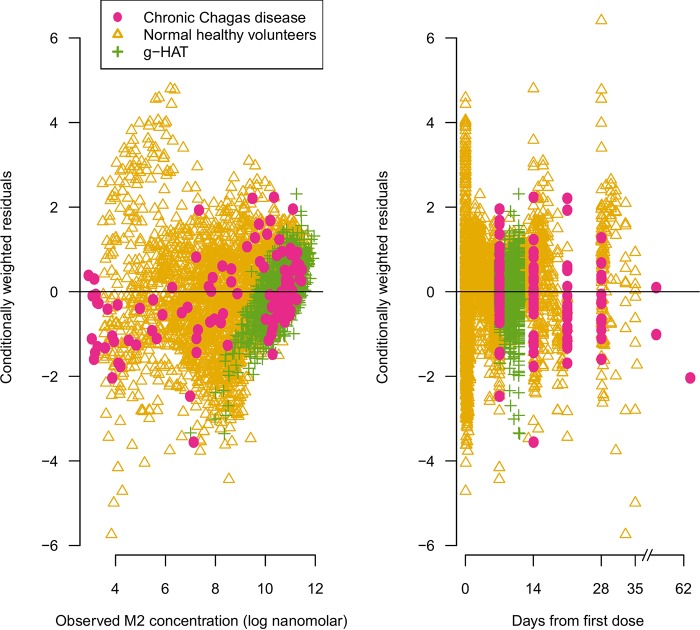
Visual pharmacokinetic model check. Comparison of the observed concentrations of the fexinidazole sulfone metabolite M2 and the conditionally weighted residuals of the final pharmacokinetic model fit to all M2 data from phase 1 trials, *g*-HAT sleeping sickness trials, and chronic Chagas disease trials. In all plots, we take as convention that hues of pink and red refer to data and summaries from the Chagas study, hues of green refer to data and summaries from the *g*-HAT studies, and hues of yellow and orange refer to data and summaries from the phase 1 studies.

The formation of the M2 fexinidazole sulfone metabolite was modeled as a first-order absorption process. A one-compartment disposition model without interindividual random variability and with additive error (on the log scale) provided the base model. Inclusion of interindividual random variability in pharmacokinetic parameters provided a significant improvement. Inclusion of one and two transit compartments for the formation of the metabolite M2 also provided significant improvement to model fits, as evidenced by the conditionally weighted residual plots. A two-compartment disposition model did not provide an improved fit to the data, as quantified by a nonsignificant improvement in the objective function. The final model was, thus, a one-compartment disposition model with two transit compartments for the formation of the metabolite. This structural model estimated that metabolite M2 had a mean transit time of 5.65 h (interindividual variability [IIV], 14%), an apparent volume of distribution of 80 liters (IIV, 20%), and an oral clearance of 3 liters/h (IIV, 14%). The full set of estimated parameters is given in Table S1 in the supplemental material. Conditionally weighted residual plots for the final model are shown in Fig. 1.

**FIG 2:**
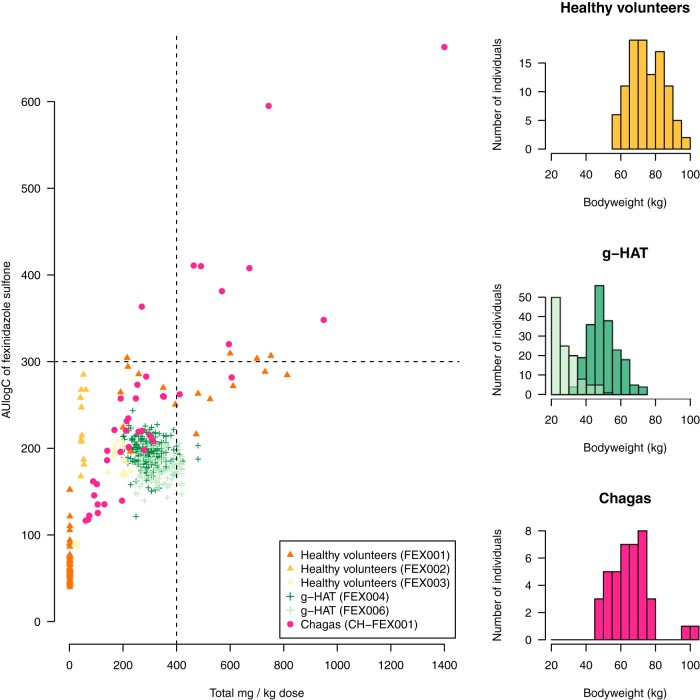
Total milligram-per-kilogram dose, body weight, and total exposure to fexinidazole sulfone across the pooled studies. (Left) Relationship between total milligram-per-kilogram dose and AUlogC for the sulfone metabolite. Colors and shapes correspond to the different studies. The horizontal dashed line shows the estimated EC_50_ for the neutropenia drug effect. The vertical dashed line shows our proposed total milligram-per-kilogram safety threshold. (Right) Distribution of body weight across the six studies. *g*-HAT patients had a lower average body weight than the NHVs or the chronic Chagas patients.

There was substantial variability in the range of total fexinidazole doses received across the six studies. The relationship between the total milligram-per-kilogram dose received and the estimated exposure to the sulfone metabolite (area under the log plasma concentration-time curve [AUlogC]) is shown in Fig. 2.

### Liver toxicity in fexinidazole-treated chronic Chagas disease patients.

**FIG 3:**
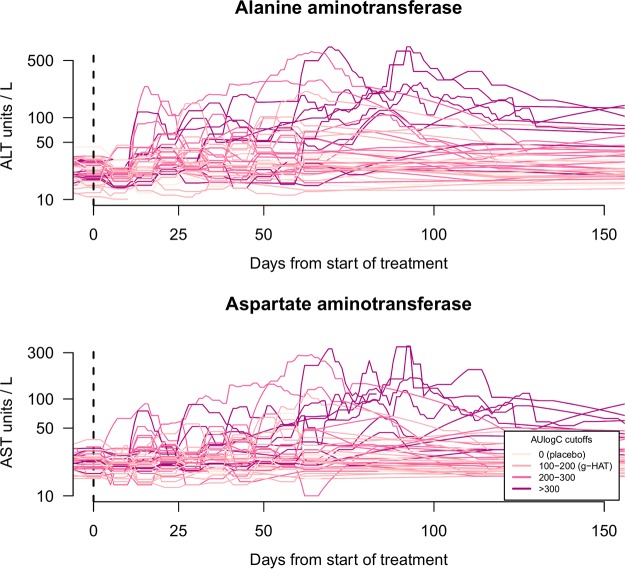
Time series data of the liver transaminases in all enrolled chronic Chagas patients. (Top) ALT; (bottom) AST. The figure shows data for all patients (*n* = 47) enrolled in the dose-finding trial of oral fexinidazole for the treatment of chronic Chagas disease. The absolute concentrations are shown on a log_10_ scale. In both panels, the color intensity corresponds to the individual total drug exposure, as quantified by the cumulative AUlogC of the M2 (sulfone) metabolite. The start of treatment (day 0) is shown by the vertical black dashed lines.

In the dose ranging study of fexinidazole for the treatment of chronic Chagas disease, dose-dependent elevations in aspartate aminotransferase (AST) and alanine aminotransferase (ALT) were observed in a subset of patients. The time series data of the liver transaminase elevations over the first 150 days following the start of treatment showed both considerable interindividual variability and heteroscedasticity but a clear separation between patients with high and low M2 exposures (Fig. 3). Twelve out of 40 (30%) chronic Chagas disease patients who received fexinidazole had ALT concentrations above 3 times the upper limit of normal (ULN); 6 out of 40 (15%) had AST concentrations above 3 times the ULN. Peak elevations of ALT and AST were observed between 50 and 100 days after the start of treatment. For the patients whose levels rose above 3 times the ULN (for males, 53 and 46 units/liter for ALT and AST, respectively; for females, 40 and 39 units/liter for ALT and AST, respectively), the duration of elevated transaminases varied between 3 and 168 days for ALT and 8 and 40 days for AST. The extent and the duration of AST and ALT elevations were explained by total drug exposure (Fig. S1). All patients’ values eventually returned to normal.

### Exposure-dependent effect of fexinidazole on hepatic transaminases.

**FIG 4:**
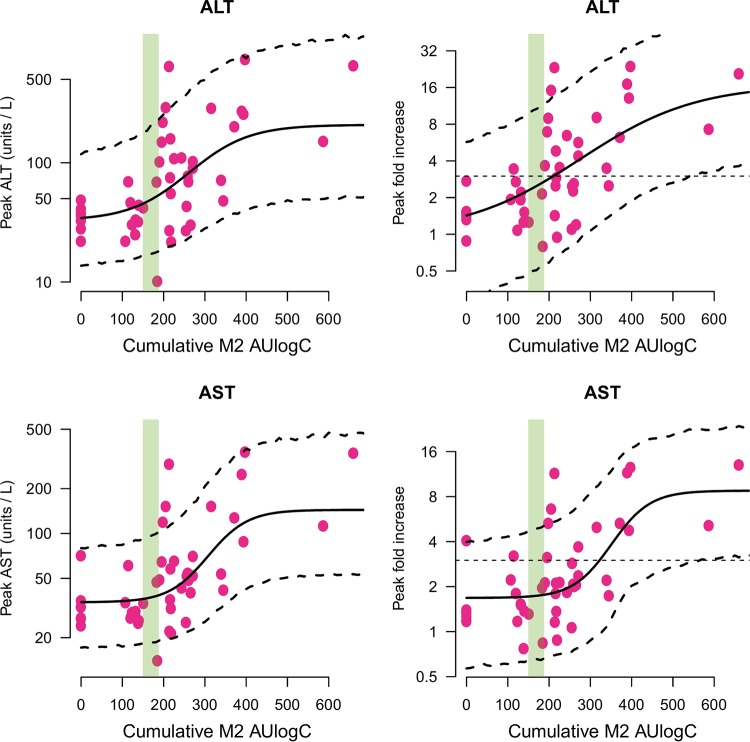
Exposure-response relationships for the peak liver transaminase values in fexinidazole-treated chronic Chagas disease patients. (Top) ALT; (bottom) AST. (Left) Relationship between fitted M2 AUlogC values and the peak observed transaminase concentrations (the *y* axis is on the log_10_ scale). (Right) Relationship between fitted sulfone metabolite (M2) AUlogC values and peak observed fold changes from the baseline (the *y* axis is on the log_2_ scale). Individual data points are shown by the pink dots; sigmoid model mean fits along with 90% posterior prediction intervals are shown by the thick and dashed lines, respectively; the range of AUlogC exposures with the *g*-HAT regimen is shown by the shaded green area. In the right column, the straight dashed black lines show the threshold value of 3 times the baseline value.

For both ALT and AST, the posterior model fits indicated clear exposure-response relationships when the pharmacodynamic outcome was measured both as absolute peak increases (Fig. 4, left) and as relative fold increases (Fig. 4, right). As quantified by the overlapping coefficient (OVL), the posterior distributions gave negligible probability (less than 5%) to the null models of no exposure-dependent outcome in all four models (Fig. S3).

For ALT, the 80% credible intervals of the marginal posterior distribution over the 50% effective dose (ED_50_) overlapped the exposure intervals in the *g*-HAT regimen (Table 2). For AST, the 80% credible intervals of the marginal posterior distribution over the ED_50_ were above the exposure intervals in the *g*-HAT regimen (Table 2). The relationship between AUlogC and the milligram-per-kilogram dose in the chronic Chagas disease trial is shown in Fig. S1.

**TABLE 2:** Summary of results from the Bayesian exposure-response models

Characteristic	Value(s) from^*a*^:			
	Absolute model		Relative model	
	EC_50_ CI_80_^*b*^	OVL^*c*^	EC_50_ CI_80_	OVL
Hematology parameters				
Neutrophils	262–342	**0**	208–352	**1**
Platelets	146–240	**3**	199–240	**0**
Lymphocytes	74–1,304	69	163–396	11
Hemoglobin	73–1,383	75	137–842	29
Liver function tests				
ALT	164–362	**2**	119–371	**0**
AST	244–371	**0**	256–396	**0**

a Absolute models evaluate changes to the absolute values of the outcomes, and relative models evaluate fold changes from the baseline in the values of the outcomes. Numbers in bold correspond to those with reasonable evidence of an exposure (dose) effect.

b EC_50_ CI_80_, 80% credible intervals over the EC_50_ values estimated by the pharmacokinetic-pharmacodynamic models.

c The overlapping coefficient expressed as a percentage (a value of 0 indicates no overlap, implying a definite exposure-response relationship; a value of 100 indicates complete overlap, implying no exposure-response relationship).

### Predicting liver toxicity in the *g*-HAT treatment regimen.

**FIG 5:**
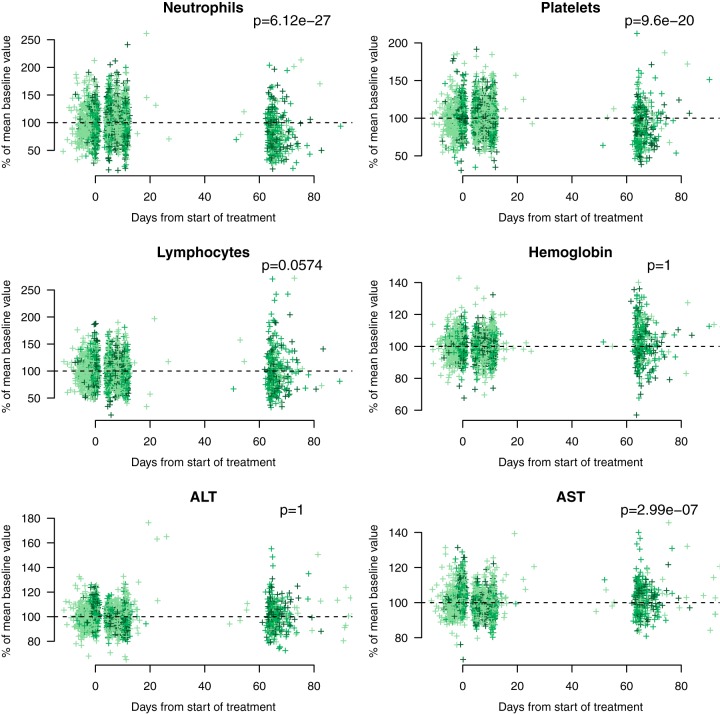
Pharmacodynamic outcomes in the *g*-HAT field trials. Each panel is a scatter plot of pharmacodynamic outcomes, shown as the percent relative change from the baseline mean value as a function of time from the start of treatment for all three field trials of fexinidazole for the treatment of *g*-HAT (T. b. gambiense). The baseline is estimated as the average of all values taken up to day 20 after the start of treatment. Shades of green correspond to the different *g*-HAT clinical trials. *P* values were computed from a Mann-Whitney U test between the early (before day 20) and late (after day 20) groups of measurements. ALT, alanine aminotransferase concentration; AST, aspartate aminotransferase concentration.

The relative models fitted to data from chronic Chagas disease patients predicted that 50% of individuals with exposures (AUlogC) distributed as observed for the *g*-HAT regimen would have ALT elevations more than 2.8 times the baseline value (more than a 180% increase) and AST elevations more than 2 times the baseline value. However, no AST or ALT elevations greater than 2 times the baseline value were observed in any of the patients in the three field trials. Indeed, for ALT, the distribution of late measurements (all measurements were taken between days 20 and 100) was identical to that of the early measurements (*P* = 1 before and during treatment; Fig. 5, bottom left). For AST, a significant difference between early and late measurements was observed (*P* < 0.01), but none had a fold change greater than 2 (Fig. 5, bottom right). Therefore, the model based on chronic Chagas disease patients overpredicted liver toxicity substantially for both transaminases in *g*-HAT patients, indicating an effect specific to the chronic Chagas disease population.

### Hematology.

**(i) Hematological variables in fexinidazole-treated chronic Chagas disease patients.** In the dose-finding study of fexinidazole in chronic Chagas disease, 8 out of 40 patients who were assigned fexinidazole had reductions in neutrophil counts, which fell to below 1,000/μl (compared to no reductions in the placebo group). The median day of the nadir value in this subgroup was day 65 (range, day 63 to day 71). These events were temporary, with rapid recoveries. The median estimated duration of neutropenia (neutrophil count below 1,000/μl), calculated using linear interpolation between adjacent time points, was 8.5 days (range, 7 to 21 days). All these patients had fexinidazole sulfone metabolite (M2) exposures, as quantified by AUlogC values, above 300 (i.e., exposures considerably greater than those seen in *g*-HAT patients; Fig. 2).

**FIG 6:**
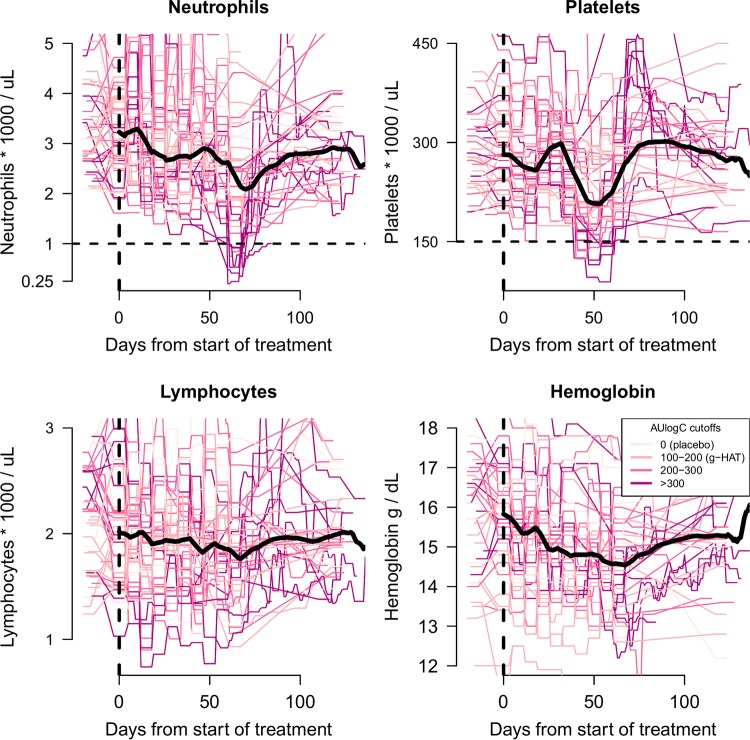
Time series data of hematological variables for all enrolled chronic Chagas disease patients. The start of treatment (day 0) is shown by the vertical black dashed lines. Colors correspond to the individual total fexinidazole exposure (AUlogC). The median trend for each hematological variable is shown by a thick black line. The threshold values of 1,000 neutrophils per μl and 150,000 platelets per μl are shown by the horizontal black dashed lines.

In addition to these more extreme variations, there was a consistent decrease in the median neutrophil counts over the course of study. This trend began from the start of treatment up until the population nadir on day 70 in all patients not receiving placebo treatment (Fig. 6, top left).

The overall profile was one of a steady decrease, with a greater reduction at about 2 months after starting treatment. Day 57 was the median day of the observed nadir in fexinidazole-treated individuals, and day 70 was the day for the population median nadir value. These decreases were transient, and by day 100 all patients had recovered. The median neutrophil and platelet counts had also reached pretreatment levels by day 100.

The time series data for platelet counts showed a temporal trend similar to that described above, albeit with a less marked initial reduction and a more marked later reduction (Fig. 6, top right). All platelet counts remained above 50,000/μl, but 9 fexinidazole-treated individuals had nadir counts below 150,000/μl, and 5 of these individuals were also in the neutropenia subgroup. The nadirs of neutrophil and platelet counts were correlated significantly (ρ = 0.5; 95% confidence interval = 0.2 to 0.7; *P* = 0.003). Nadir platelet counts were seen approximately 2 weeks before nadir neutrophil counts. The median observed day of the nadir for the platelet counts in fexinidazole-treated patients occurred on day 44; the population nadir median value was on day 53. In patients with platelet counts below 150,000/μl, the median estimated duration of thrombocytopenia, calculated using linear interpolation between adjacent time points, was 9 days (range, 5 to 23 days).

Median hemoglobin counts also dropped by 1.3 g/dl by day 67, the day for the population median nadir value (Fig. 6, bottom right). In contrast, lymphocyte counts showed no clear trend (Fig. 6, bottom left).

**FIG 7:**
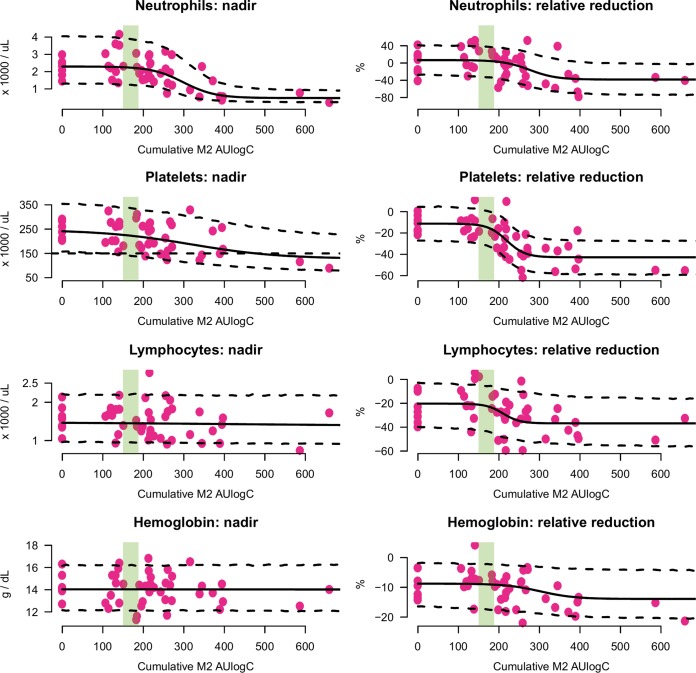
Exposure-response relationships for the main hematological variables of interest in the dose-finding assessment of fexinidazole in chronic Chagas disease patients. Exposure is quantified by the total cumulative M2 metabolite AUlogC. (Left) Scatter plots between fitted exposures and nadir observed values (pink dots). (Right) Scatter plots between M2 AUlogC exposure and the maximum observed relative percent decrease with respect to the individual baseline value. Sigmoid model mean fits along with 90% posterior prediction intervals are shown by the thick and dashed lines, respectively. The range of fitted drug exposures after the *g*-HAT regimen is shown by the shaded green areas.

**(ii) Hematological exposure-response effects of fexinidazole.** For both neutrophil and platelet counts, the models indicated clear exposure-response relationships when the pharmacodynamic outcome was measured both as absolute peak increases (Fig. 7, left) and as relative fold increases (Fig. 7, right). As quantified by the overlapping coefficient, the posterior distributions gave negligible probability (less than 5%) to the null model of no exposure-dependent outcome in all four models (Fig. S3). For lymphocyte counts and hemoglobin concentrations, there was no clear evidence of an exposure-response effect, with OVL coefficients varying between 11% and 75%.

For reductions in neutrophil counts, the credible intervals over the 50% effective concentration (EC_50_) parameters in both the absolute and the relative models were above the *g*-HAT patient exposures (Table 2). However, for the reductions in platelet counts, the absolute model suggested an EC_50_ within the *g*-HAT patient exposures (Table 2).

### Predicting drug effects on hematological variables in *g*-HAT patients.

For exposures distributed according to those observed in the *g*-HAT regimen, the relative models trained on the data from the chronic Chagas disease patients predicted a median relative decrease in neutrophil counts of 0%, with 10% of patients being predicted to have relative reductions of approximately 35%. For platelet counts, the predictions were for a median decrease of 20%, with 10% experiencing decreases of more than 35%. Statistically significant but clinically insignificant decreases in both neutrophil and platelet counts from the baseline were observed in *g*-HAT patients (*P* < 0.001 for both; Fig. 5, top two panels).

The median decreases were 20% and 10% for neutrophils and platelets, respectively. The 90th percentiles for neutrophils and platelets were 60% and 40%, respectively. The timing of the late full blood counts done in the *g*-HAT trials coincided with the timing of the population nadir for neutrophil counts but not that of the population nadir for platelet counts (2 weeks earlier) in the chronic Chagas disease trial. This difference in the timing of hematological changes was not known at the time of the *g*-HAT trial protocol amendments.

**FIG 8:**
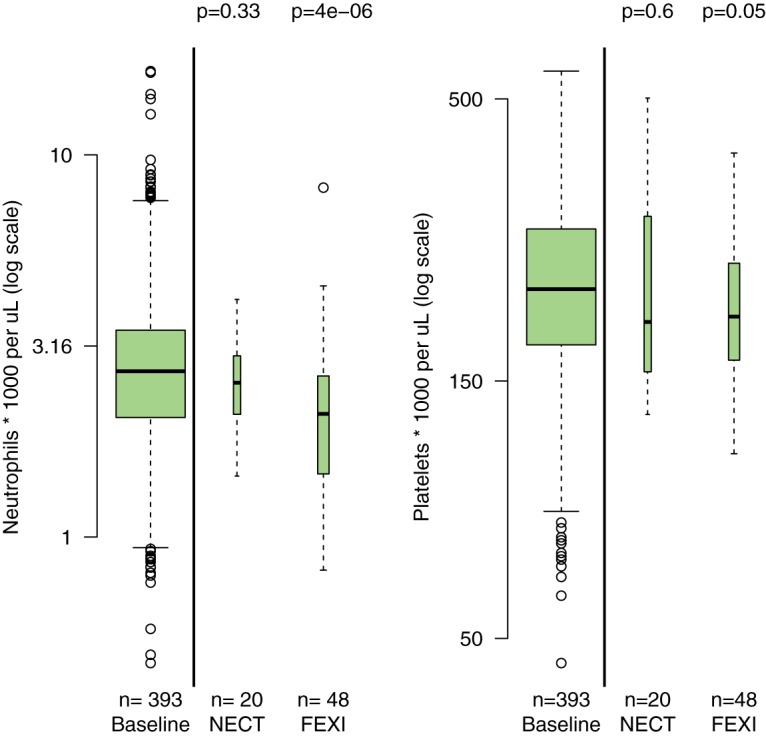
Comparison between early (baseline and during treatment) and late (circa day 70) neutrophil (left) and platelet (right) counts grouped by randomized treatment in trial FEX004 (adults with late-stage *g*-HAT). *P* values were computed from a Mann-Whitney U test between early (all patients) and late measurements. The width of each box-and-whisker plot is proportional to the square root of the number of observations. The *y* axes in both panels are on a log_10_ scale. FEXI, fexinidazole; NECT, nifurtimox-eflornithine.

These observed reductions in neutrophil and platelet counts could be confounded. Patients in the chronic Chagas disease study were asymptomatic, whereas *g*-HAT patients were ill and, thus, may have had higher neutrophil and platelet counts on admission. This possible confounding effect can be approximated by comparing the observed decreases in the *g*-HAT patients receiving fexinidazole and those receiving nifurtimox-eflornithine combination therapy (NECT) in the FEX004 study (randomized assignment). For both platelet and neutrophil counts, there were no significant reductions in the NECT group, whereas there were in the fexinidazole group (Fig. 8).

Although the numbers were small (*n* = 20 late counts in the NECT arm), this suggests that the fexinidazole regimen for *g*-HAT treatment results in mild but predictable delayed reversible decreases in neutrophil and platelet counts.

## DISCUSSION

Fexinidazole will likely replace nifurtimox-eflornithine as the treatment of choice for human African trypanosomiasis caused by Trypanosoma brucei gambiense (*g*-HAT) (1–4, 10). In large, controlled studies, it has proved well tolerated and effective (the findings from the FEX004 trial are reported in reference 11, and findings from the FEX005 and FEX006 trials are as yet unpublished). A safe, once-daily, oral treatment substantially improves the prospects for elimination of this major tropical neglected disease. The clinical results in patients with *g*-HAT and the significant *in vitro* activity of fexinidazole against other kinetoplastid parasites prompted investigations in patients with visceral leishmaniasis and Chagas disease, but the preliminary investigations in patients with chronic indeterminate Chagas disease were interrupted when some patients developed marked but transient delayed neutropenia. In addition, significant elevations in aspartate and alanine aminotransferases were noted, suggesting liver toxicity. These adverse reactions were unusual, in that they sometimes occurred up to 2 months after starting the fexinidazole treatment, often well after the drug had been stopped and the bioactive parent and metabolites would have been cleared.

Fexinidazole is rapidly converted *in vivo* by oxidative metabolism involving several different cytochrome P450 enzymes to sulfoxide and sulfone metabolites which retain biological activity (5), but the majority of bioactive exposure *in vivo* is to the sulfone metabolite (M2). With the available data, it is not possible to dissociate the toxicity relationships of the parent compound and metabolites, and so the only associations explored here are with the sulfone metabolite. The mechanisms of fexinidazole toxicity are not known but appear to be class effects, although why neutrophil and platelet counts fall approximately 9 and 7 weeks after starting treatment, respectively, and in many cases weeks after completing fexinidazole treatment is not known. The sequential timing of neutrophil and platelet reductions and their correlation without coincidental lymphopenia suggest bone marrow suppression. The transient nature of these reductions (lasting approximately 1 week) suggests a transient inhibition or suppression of a bone marrow precursor.

This pharmacokinetic-pharmacodynamic assessment used total cumulative log concentrations (total AUlogC) of metabolite M2 as a proxy measurement of drug exposure. This is justified for several reasons. The first is the ease of estimation, as M2 is formed slowly and has the most predictable pharmacokinetic behavior with the least interindividual variability. Thus, in the dose assessment trial of fexinidazole in chronic Chagas disease patients, which provided the large majority of the pharmacodynamic information, sparse drug measurements taken over 8 weeks can be summarized reliably by model estimates of the M2 AUlogC. Second, M2 concentrations have been shown to be the best predictor of electrocardiograph QT prolongation (unpublished observations) and vomiting. The relationship between the total milligram-per-kilogram dose of fexinidazole and the pharmacokinetic exposure is imprecise for higher doses but gives an approximate threshold dose of 400 mg/kg of body weight for a threshold AUlogC exposure of 300 (Fig. 2).

The nitroimidazoles are a class of drugs with a known potential for both neutropenia and liver toxicity (12–17), although cholestatic hepatitis is the usual manifestation (18). One patient in the phase 1 studies of fexinidazole showed evidence of hepatotoxicity on day 15 after 14 daily doses of 3,600 mg administered under fasted conditions. The values spontaneously returned to normal (7). No case meeting Hy’s law criteria was reported. However, follow-up in the phase 1 studies was short (a maximum of 28 days after the start of the regimen), so the possibility of later asymptomatic hepatotoxicity following these studies cannot be excluded. The duration of the dose regimens of fexinidazole evaluated in chronic Chagas disease patients was largely based on the regimen of benznidazole, along with some limited pharmacokinetic-pharmacodynamic modeling of mouse data. Benznidazole, another nitroimidazole, is the currently recommended treatment of Chagas disease and has also been associated with both hepatotoxicity and neutropenia (19). Benznidazole is usually given for 8 weeks, and so in some patients, the total milligram-per-kilogram dosing of fexinidazole was substantially higher than that in the 10-day *g*-HAT regimen (in one arm, it was 7 times higher). The principal findings of this retrospective pharmacokinetic-pharmacodynamic study are that the risks of increased transaminases, delayed neutropenia, and delayed platelet reductions are proportional to drug exposure but that exposures in *g*-HAT patients are below those associated with clinically significant toxicity. The estimated EC_50_, quantified in terms of the total M2 AUlogC for hepatoxicity and reductions in neutrophil and platelet counts, is considerably higher than the maximum observed M2 AUlogC values in patients treated for *g*-HAT.

The observed reductions in neutrophil and platelet counts in the field trials of fexinidazole in *g*-HAT patients match predictions from the pharmacokinetic-pharmacodynamic model built using the data from the dose assessment trial in chronic Chagas disease patients. These were mild and clinically insignificant reductions in neutrophil and platelet counts, which are very unlikely to pose a threat to *g*-HAT patients. On the other hand, the predicted dose-dependent hepatotoxicity based on the chronic Chagas disease patient data was not observed in the treatment of *g*-HAT patients by fexinidazole. This suggests an additional disease effect which is specific to Chagas disease or to the Bolivian population studied. Future studies of fexinidazole for the treatment of Chagas disease need to have long follow-up (4 to 6 months) in order to assess potential iatrogenic changes to hematological variables and liver transaminases.

### Conclusion.

Taken together these data suggest that there is a satisfactory margin of safety for dose-related toxicity with the current fexinidazole regimen for the treatment of *g*-HAT. Future trials of fexinidazole in Chagas disease patients should assess liver function over a period of at least 4 months following the start of treatment. Shorter regimens of fexinidazole (less than 10 days) should be safe for the treatment of chronic Chagas disease. Transient but clinically significant neutrophil decreases are to be expected in individuals taking a total dose of more than 400 mg/kg of oral fexinidazole.

## MATERIALS AND METHODS

### Clinical trials.

In all of the clinical trials of fexinidazole reported here, healthy volunteers, patients, or their guardians provided full informed consent. Further details of the ethical reviews can be found at the ClinicalTrials.gov website using the reference registration numbers provided below.

### Phase 1 studies in normal healthy volunteers.

Three separate phase 1 studies were carried out in 116 normal healthy male volunteers (NHV) to assess the tolerability of oral fexinidazole and to characterize its pharmacokinetics. Full details of these phase 1 studies and results from noncompartmental pharmacokinetic analyses are reported elsewhere (7). Electrocardiographic recordings were collected in all trials, but these data are being analyzed separately.

**(i) FEX001.** FEX001 was a randomized, double-blind, placebo-controlled study of the tolerability and the pharmacokinetics of oral fexinidazole given in single and repeated doses (ClinicalTrials.gov registration number NCT00982904). This study also included a comparative bioavailability study of an oral suspension versus the tablet formulation and an exploratory assessment of food effects. All subjects were healthy male volunteers of sub-Saharan African origin. The pharmacokinetics of oral fexinidazole were characterized in three separate substudies. Dense pharmacokinetic sampling was performed in all substudies at the following time points: predose and 0.5, 1, 2, 3, 4, 6, 9, 12, 16, 24, 48, 72, 96, 120, 144, and 168 h postdose. Part 1 consisted of single doses ranging from 100 to 3,600 mg (*n* = 54). Part 2 consisted of testing for bioequivalence between the oral tablet form and the oral suspension form, which was not included in this analysis (*n* = 11). Part 3 consisted of daily dosing for 14 consecutive days with doses of 1,200, 2,400, and 3,600 mg (*n* = 17).

**(ii) FEX002.** FEX002 was a randomized, open-label study to assess the effect of two different types of food conditions versus the fasted condition on the relative bioavailability of a single dose of oral fexinidazole in healthy males (*n* = 12) (ClinicalTrials.gov registration number NCT01340157). Plasma concentration (total and free) measurements were taken at 5 nominal time points (1, 4, 12, 24, and 72 h postdose). Frequent venous sampling was done at the same time points used for the FEX001 study.

**(iii) FEX003.** FEX003 was a randomized, double-blind, placebo-controlled comparison of two 10-day regimens of fexinidazole (ClinicalTrials.gov registration number NCT0148370), with both including a 4-day loading dose. Regimen 1 was administered as 1,800 mg for 4 days, followed by 1,200 mg for 6 days. Regimen 2 was administered as 2,400 mg for 4 days, followed by 1,200 mg for 6 days. Each dose was taken after a meal. All subjects (*n* = 22 subjects completed the study out of 30 included subjects) were healthy male volunteers, with both parents of each volunteer being of sub-Saharan African origin. Pharmacokinetic samples were taken on days 1, 4, and 7 at the following nominal time points: predose and then at 0.5, 1, 2, 3, 4, 6, 9, 12, 16, and 24 h postdose. On day 10 (last dose), pharmacokinetic samples were taken predose and then at 0.5, 1, 2, 3, 4, 6, 9, 12, 16, 24, 48, 72, 96, 120, 144, and 168 h postdose.

### Treatment trials in patients with T. b. gambiense sleeping sickness (*g*-HAT).

The phase 2/3 trial (FEX004) was conducted in the Democratic Republic of Congo (DRC) and Central African Republic (CAR). This pivotal study was conducted in 394 adult stage 2 *g*-HAT patients (i.e., in patients with CNS involvement). Two additional cohort studies (FEX005 and FEX006) were conducted in 230 patients with stage 1 *g*-HAT (i.e., no CNS involvement) and 125 children with both stages of *g*-HAT who were aged 6 to 14 years and weighed more than 20 kg. All patients had to have parasitologically confirmed *g*-HAT and a Karnovsky score of *>*50 in order to be eligible for enrollment. Adult patients and children weighing more than 35 kg were treated with fexinidazole at 1,800 mg once daily for 4 days, followed by 1,200 mg once daily for 6 days. Children older than 6 years and weighing between 20 and 35 kg were given an adapted regimen: 1,200 mg for 4 days, followed by 600 mg for 6 days. All field trials administered fexinidazole as 600-mg tablets in blister packaging.

**(i) FEX004.** FEX004 was the pivotal phase 2/3 study assessing the safety and efficacy of fexinidazole in the treatment of *g*-HAT (ClinicalTrials.gov registration number NCT01685827) (11). It was an open-label randomized trial of oral fexinidazole compared to the current standard-of-care regimen of nifurtimox-eflornithine combination therapy (NECT) in adult patients (*>*15 years old) with late-stage *g*-HAT. Late-stage sleeping sickness is defined as confirmed trypanosome parasites in the cerebrospinal fluid (CSF) or confirmed parasites in blood and a CSF white cell count greater than 20/μl. The randomization ratio was 2:1, with 264 patients enrolled into the fexinidazole arm and 130 patients enrolled into the NECT arm. The NECT regimen was a combination of oral nifurtimox tablets at 5 mg/kg three times daily for 10 days (days 1 to 10) and eflornithine at 200 mg/kg administered twice daily as a 2-h intravenous infusion for 7 days. The fexinidazole adult regimen was 4 daily doses of 1,800 mg, followed by 6 daily doses of 1,200 mg. This is referred to as the *g*-HAT regimen. The study took place at 10 sites in the Democratic Republic of Congo and the Central African Republic.

Pharmacokinetic samples were taken from 203 out of 264 (77%) of the fexinidazole-treated patients. The field sites were very remote, and it was not possible to store frozen samples on-site. Capillary whole blood was therefore collected and aliquoted onto filter paper to produce dry blood spots (DBS; 300 μl [20]) from a finger-prick sample at the following time points: on day 8 at 3 h after the dose, on day 9 at 3 h after the dose, on day 10 at 3 h and 7 h after the dose, and on days 11 and 12 at 24 and 48 h after the last dose. A lumbar puncture was performed on day 11 (24 h after the final dose) for the efficacy assessment. Due to technical difficulties (the majority of patients refused the additional lumbar puncture), in 82 out of the 264 (31%) fexinidazole-treated patients, an aliquoted (300-μl) CSF sample from the follow-up lumbar puncture was allowed to dry on filter paper and stored for later drug measurement. Full blood counts and biochemistry data were taken at enrollment and then on days 5 and 11 after the start of treatment. In response to the hematology and biochemistry abnormalities recorded during the chronic Chagas disease treatment trial, the protocol was modified so that all patients not lost to follow-up (68 out of 264, 26%) could have full blood counts and biochemistry data checked 9 weeks after the start of treatment.

**(ii) FEX005.** FEX005 was the first plug-in study (i.e., identical clinical sites, clinical trial staff, patient population, and fexinidazole regimen), which included stage 1 and early stage 2 adult *g*-HAT patients receiving the same fexinidazole regimen used in FEX004 (ClinicalTrials.gov registration number NCT02169557). It was an open-label single-group study which enrolled 230 patients. No pharmacokinetic sampling was carried out, but full blood counts and biochemistry data were taken at enrollment and then on days 5 and 11 and at 9 weeks following the start of treatment.

**(iii) FEX006.** FEX006 was the second plug-in study of fexinidazole in children older than 6 years old and weighing more than 20 kg with stage 1 and 2 *g*-HAT (ClinicalTrials.gov registration number NCT02184689). Children weighing between 20 and 35 kg were given 1,200 mg of fexinidazole daily on days 1 to 4 (2/3 of the adult dose) and then 600 mg daily on days 5 to 10 (half the adult dose). Children weighing more than 35 kg were given the adult regimen. This analysis included pharmacokinetic data from 114 patients. A series of pharmacokinetic measurements using DBS was taken on days 10 (3 and 7.25 h after the final dose), 11 (24 h after the final dose), and 12 (48 h after the final dose). Drug measurement was also performed on dried CSF on filter paper (as in FEX004) from the day 11 lumbar puncture in the first 30 patients. Full blood counts and biochemistry data were taken at enrollment and then on days 5 and 11 and at 9 weeks after the start of treatment.

### Treatment trial in chronic Chagas disease patients.

A dose-finding study (ClinicalTrials.gov registration number NCT02498782) in adult Bolivian patients with chronic indeterminate Chagas disease (here referred to as chronic Chagas disease) began in July 2014. Nonpregnant adult patients were enrolled if they were positive for a validated T. cruzi PCR test but had no clinical evidence of end organ damage. The duration of treatment was structured around currently recommended regimens for the treatment of T. cruzi infections with benznidazole. All patients were outpatients. Patients were advised to take the treatment as a single daily dose and with a meal. Each week they were given enough medication until the next scheduled weekly visit. Treatment was thus unobserved, and drug adherence was checked weekly by pill counting.

Benznidazole rescue treatment at the end of the study was offered for nonresponders. Patients were randomized to one of seven once-daily dosing regimens: (i) fexinidazole at 1,200 mg daily for 2 weeks, followed by matching placebos for 6 weeks; (ii) fexinidazole at 1,200 mg daily for 4 weeks, followed by matching placebos for 4 weeks; (iii) fexinidazole at 1,200 mg daily for 8 weeks; (iv) fexinidazole at 1,800 mg daily for 2 weeks, followed by matching placebos for 6 weeks; (v) fexinidazole at 1,800 mg daily for 4 weeks, followed by matching placebos for 4 weeks; (vi) fexinidazole at 1,800 mg daily for 8 weeks; and (vii) placebos for 8 weeks.

After completion, the patients were followed for 12 months. Pharmacokinetic samples were taken on day 0 (predosing), on day 1, and then weekly for weeks 2 to 5 and then on weeks 9 and 10. Laboratory hematology and biochemistry samples were taken on day 0 and weekly for weeks 2 to 10. The study was interrupted approximately 3 months later after the enrollment of 47 subjects because of the aforementioned toxicity concerns.

### Drug measurements.

Blood samples from the phase 1 studies were taken into lithium heparin tubes and immediately centrifuged, and plasma was separated and stored at −70°C until bioanalysis. Fexinidazole and its sulfoxide (M1) and sulfone (M2) metabolites were analyzed on a Supelco Ascentis Express C_18_, 2.7-μm-particle-size, 50- by 4.6-mm (inside diameter) column using a validated liquid chromatography-tandem mass spectrometry (LC-MS/MS) method. The plasma lower limits of quantification for fexinidazole, M1, and M2 were 0.5, 10, and 10 ng/ml, respectively. For clinical sampling, Whatman no. 903 Protsaver 5 spot paper, purchased from GE Healthcare Bio-Sciences (France), was used. Upon arrival of the DBS samples in the bioanalytical laboratory and before analysis, a visual inspection of the quality of the spot was performed. A blood spot was considered valid if the following criteria were met: (i) the spot diameter was equal to or greater than 7 mm, (ii) the spot was spread symmetrically on both sides of the sampling paper, and (iii) the spot was made from a single drop of blood and was dark red in color. The blood volume deposited onto the filter paper and the position of the punch had no effect on the quantitation of the test compound. Hematocrit values between 30% and 50% were shown not to affect the accuracy of drug measurement.

The ratio of capillary blood fexinidazole and M1 and M2 metabolites to simultaneous plasma concentrations stayed constant over time at 0.59, 1.00, and 0.97, respectively, regardless of the plasma concentrations of fexinidazole, M1, and M2 and the sampling time.

### Pharmacokinetic analysis.

The majority of the pharmacodynamic events of interest (evidence of hematological and liver-related toxicities) occurred in the dose-finding assessment of fexinidazole in chronic Chagas disease patients. This trial had only sparse pharmacokinetic sampling. Thus, the primary goal of the pharmacokinetic modeling exercise was to impute as reliably as possible the pharmacokinetic profiles of these patients. An unpublished internal DND*i* report shows that metabolite M2 is the primary determinant of both efficacy and physiological events (QT interval prolongation). For this reason, we used the pharmacokinetic profile of the slowly eliminated sulfone metabolite (M2) as the determinant of the exposure-related adverse events. This metabolite is also the most stable among the three compounds, exhibiting the least interindividual variability (7).

The pharmacokinetic data analysis was done using NONMEM (v.7.4) software (ICON Development Solutions, Ellicott City, MD). Molar units of metabolite M2 concentrations were transformed into their natural logarithms and modeled using both 1- and 2-compartment disposition models with first-order formation and elimination. Multiple candidate structural models were evaluated for the formation of the drug, using 0, 1, 2, or 3 transit compartments. The different structural models were evaluated on the dense data from the phase 1 studies. Scaling of parameters by weight was evaluated using the allometric relationship (weight/median weight)^0^*^.^*^75^ for clearances and a linear relationship (ratio of weight to median weight) for volumes. A food effect was also introduced as a covariate for the volume of distribution (in some phase 1 studies, the drug was given to fasting subjects, whereas in all field trials the drugs were administered after the ingestion of food). The final estimation of the model parameters used all data from the phase 1, *g*-HAT, and Chagas disease field trials. Variations between these phase 1 and treatment trials (study and disease effects) were considered only as changes to the relative absorption parameter *F* (scaling parameter on *F*). The data from both the *g*-HAT and Chagas disease field trials were too sparse to estimate changes in the absorption rate, clearance, or volume accurately. Trial-specific effects were introduced in the model as categorical covariates with a linear effect in terms of the percent reduction for both the clearance and volume parameters. Three categories were defined (NHVs, *g*-HAT trials, and Chagas disease trial). The final NONMEM model code is provided in the supplemental material.

### Pharmacodynamic and statistical analyses.

All the pharmacodynamic and statistical analyses were performed using R software (R Core Team, 2016).

### AUlogC to quantify drug exposure.

From the dose-finding study in chronic Chagas disease patients, the main pharmacodynamic events identified were delayed reductions in neutrophil counts and rises in plasma concentrations of liver transaminases. In preclinical and clinical studies, there was no evidence for very slowly eliminated metabolites. Therefore, because of the long interval between drug exposure and these pharmacodynamic outcomes (i.e., after the almost complete elimination of the drug and its active metabolites), the pharmacokinetic driver could not have been the drug concentrations at the time the adverse effects were noted but was an overall summary of exposure with hysteresis in the concentration-effect relationship. In this work, we used the total cumulative area under the log concentration-time curve (AUlogC) as the pharmacokinetic proxy for drug exposure.

### Estimating exposure-response curves.

This was a retrospective exploration of the relationships between total drug exposure and the main observed adverse events. All pharmacokinetic-pharmacodynamic models were fitted in a Bayesian framework using *stan* (21) with weakly informative priors for all parameters. Posterior distributions are shown in Fig. S2 in the supplemental material. The model code and exact prior specification are also provided in the supplemental material.

Six pharmacodynamic outcomes were examined: four hematological (nadir observed blood neutrophil counts, platelet counts, lymphocyte counts, and hemoglobin) and two related to liver toxicity (peak observed serum transaminases). The steady-state dynamics of these four hematological parameters were substantially different. In the absence of biological perturbations, the red cell counts and, thus, the blood concentrations of hemoglobin are very stable processes within an individual, with daily variations of about ±0.5 g/dl. For individuals in the chronic Chagas disease study, this corresponds to variations of approximately 3% around the baseline. Neutrophil, platelet, and lymphocyte counts exhibited much larger variations at steady state, with the baseline intraindividual variation being up to 50% in this data set. For this reason, quantifying time-dependent changes to these four processes necessitates different methodologies. For example, large variations of ±50% in neutrophil counts above a lower threshold count of 1,000*/*μl are considered normal, whereas a decrease of 10% in hemoglobin could be considered clinically relevant even when staying within the normal-range bounds. In order to account for these differences in temporal dynamics, we analyzed both the absolute values (nadir values for hematology and peak values for liver transaminases observed after the start of treatment) and the relative values (maximum relative decreases or increases from the baseline, respectively). Individual baseline values were calculated as the mean value observed in the pretreatment visit and the day 0 visit (the start of fexinidazole treatment), i.e., two values per person. For transparency, we present the results from both analyses for all pharmacodynamic outcomes. Throughout, models based on the absolute pharmacodynamic values are denoted the absolute models, and those based on the relative changes are denoted the relative models.

To estimate the exposure-response curves for the hematological parameters and the liver toxicity outcomes, we fitted four-parameter sigmoid functions, defined as follows:

(1) $$f(x)=E_{\min\;}+\frac{E_{\max\;}-E_{\min\;}}{1+e^{k\left(x-{\text{EC}}_{50}\right)}}$$

where *f* is the pharmacodynamic outcome of interest modeled as a function of drug exposure *x*, *E*_min_ is the baseline mean outcome under no or negligible drug exposures, *E*_max_ is the asymptotic maximal effect for high drug exposures, EC_50_ is the drug exposure corresponding to (*E*_max_ + *E*_min_)/2 (half-maximal-effect concentration), and *k* parameterizes the slope of this sigmoid relationship. In the *stan* specification of the model, the slope parameter *k* was explored in log space to improve convergence.

The relative models (which can have both positive and negative outcome values) use a normal additive error term parameterized by its standard deviation, σ_add_. The absolute models (only positive outcomes) use a proportional normal error term, σ_prop_: log(*y*) ∼ *N*[*f*(*x*), σ_prop_], where *N* is the normal distribution, with mean *f*(*x*) the sigmoid regression mean prediction and standard deviation σ_prop_, and *y* is the observed outcome. The pharmacodynamic models were fitted using all available data from patients with chronic Chagas disease.

These patients received the largest variety of total milligram-per-kilogram doses and had the richest observed pharmacodynamic outcomes. Individuals in the phase 1 studies also received high total doses of fexinidazole, but follow-up was only 28 days, and therefore, the outcome data are not directly comparable. For this reason, we used only the data from the chronic Chagas disease patients to fit the pharmacokinetic-pharmacodynamic models.

### *Post hoc* evaluation of an exposure-response relationship.

This was a *post hoc* pharmacokinetic-pharmacodynamic analysis and, as such, was prone to data-dependent analyses and false-positive results (22). We attempted to minimize this danger by avoiding an analysis contingent on significant *P* values of a null hypothesis that there was no exposure-response relationship (which can be difficult to control properly for multiple comparisons) and instead summarized the posterior evidence for exposure-dependent pharmacodynamic outcomes.

The null model for equation 1 is one for which there is no exposure-dependent pharmacodynamic outcome and can be defined as the model for which *E*_min_ is equal to *E*_max_. Thus, the model collapses to a simple zero-order linear trend (slope coefficient, 0). The posterior evidence of the null model is quantified by the overlap between the marginal posterior distributions over *E*_max_ and *E*_min_. This overlap is quantified by the overlapping coefficient, defined as a distance metric between two densities, *f* and *g*, with the same support *x* in *Z* (23):

(2) $$\text{OVL}\left(f,g\right)\;=\int_{z}\text{min}\left[f(x),g(x)\right]dx$$

This is an intuitive measure of the overlap between two arbitrary densities of the same support, *f* and *g*, with values varying between 1 (complete agreement) and 0 (complete disagreement).

### Predicting toxicity in the fexinidazole regimen recommended for *g*-HAT.

The trial of fexinidazole in chronic Chagas disease had regular full blood counts (weekly up to week 10 and then monthly), providing reasonable confidence that the observed time series patterns for hematological parameters and liver function are indicative of the true underlying patterns. For the three *g*-HAT field trials, weekly blood counts in the weeks following treatment were not performed. Some late measurements were taken in FEX004 (adults with late-stage *g*-HAT), and most patients in the FEX005 and FEX006 trials had one late measurement (at about week 10). Thus, the distribution of nadir relative decreases for neutrophil counts is expected to be different between the two studies because of the trial design. Because of the biases introduced by these different trial designs and the differences in the two populations (for example, individuals of sub-Saharan African descent have lower neutrophil counts due to different genetic variants of the Duffy gene [24]), we cross predicted using only the relative models (nadir or peak values divided by the baseline value).

## Supplementary Material

Supplemental file 1
