# Supplementary material for: Pharmacokinetic-Pharmacodynamic Assessment of the Hepatic and Bone Marrow Toxicities of the New Trypanoside Fexinidazole
Source: Antimicrob Agents Chemother. 2019 Mar 27;63(4):e02515-18. doi: 10.1128/AAC.02515-18 (PMC6496162; doi:10.1128/AAC.02515-18)
Supplement: Supplemental file 1 [file AAC.02515-18-s0001.pdf]

Supplementary materials for: ‘A pharmacokinetic-pharmacodynamic assessment of the hepatic and bone-marrow toxicities of the new trypanoside fexinidazole’

James A Watson[1,2*], Nathalie Strub-Wourgraft[3], Antoine Tarral[3], Isabela Ribeiro[3], Joel Tarning[1,2], Nicholas J White[1,2*],

1 Supplementary figures

Figure S1: The duration of chronic elevated liver transaminases in Chagas disease patients

Figure S2: Posterior distributions over the ED_{50} values for the fitted pharmacokinetic-pharmacodynamic relationships

Figure S3: Posterior distributions over the $E_{\min}$ (thick lines) and $E_{\max}$ (dashed lines) values for all models and for all pharmacodynamic outcomes of interest

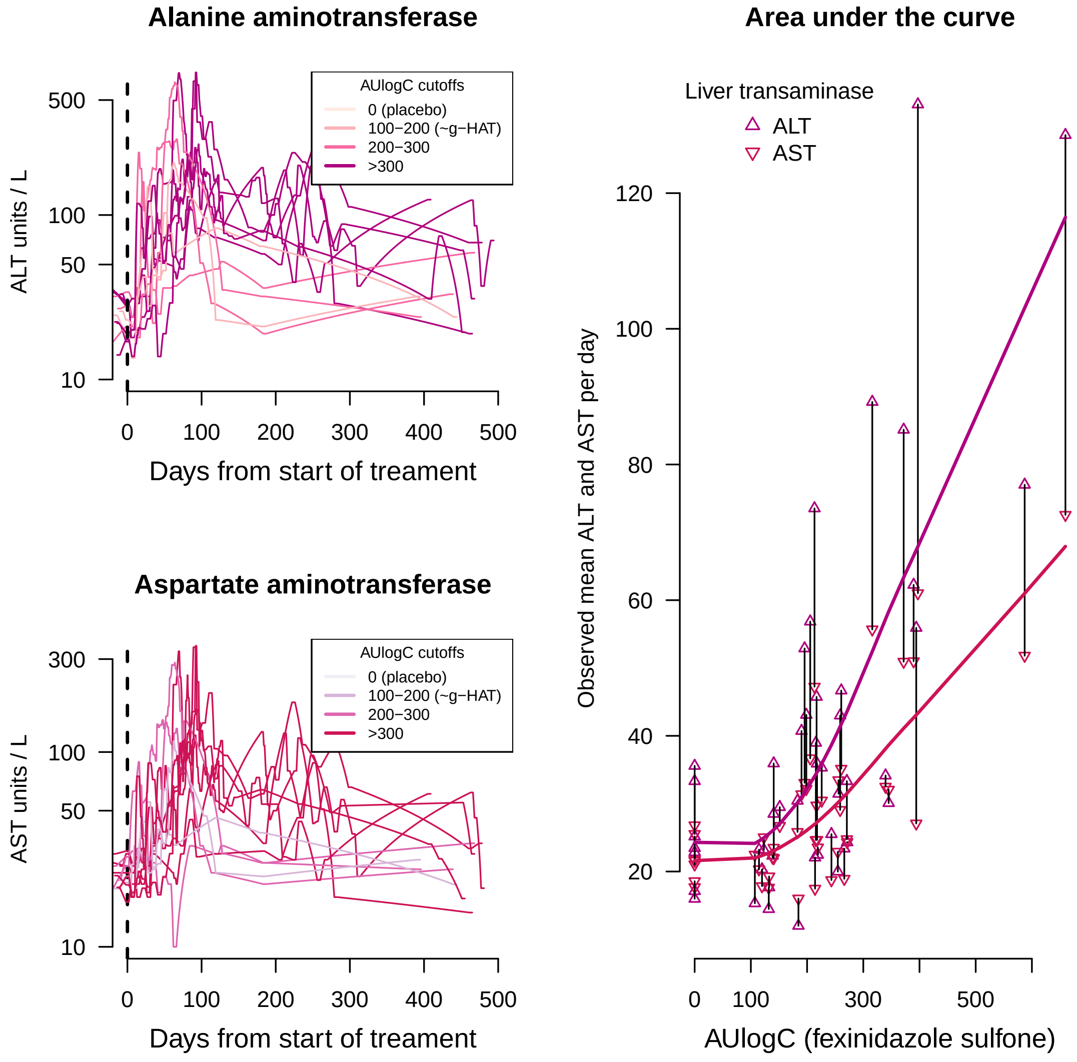

Figure 1: **The duration of chronic elevated liver transaminases in Chagas disease patients.** The two left panels show the time-series data for alanine (ALT: top) and aspartate (AST: bottom) for all patients who had values above 3X the upper limit of normal (gender dependent value). Follow-up in these patients was extended up to 500 days after the start of treatment. The right panel shows the relationship between pharmacokinetic exposure to the fexinidazole sulfone metabolite and the average daily area under the curve (AUC) for the ALT (purple upward pointing triangles) and AST (red downward pointing triangles) concentrations in all patients over the course of follow-up. Black lines connect distinct AUCs for AST and ALT within the same individuals. Spline fits (R function *loess* with *span* = 1) are shown by the thick lines to indicate the overall trend in the data

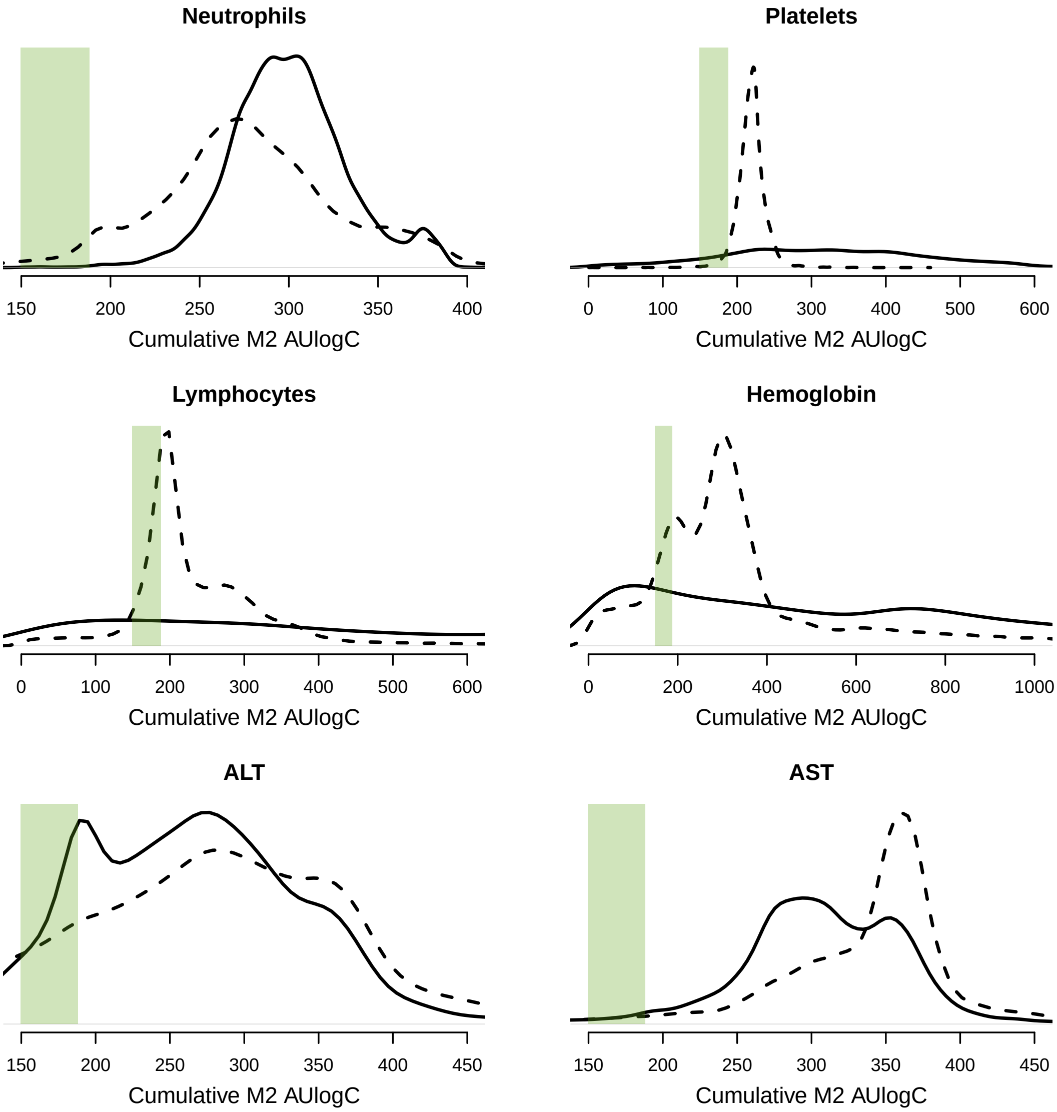

Figure 2: **Posterior distribution of ED_{50} values for the fitted PK-PD relationships**. The shaded green areas show the ranges of exposures observed in the g-HAT regimen. The black lines show the posterior distributions for each model. Dashed lines show the posterior distributions for the *relative* models and the thick lines for the *absolute* models.

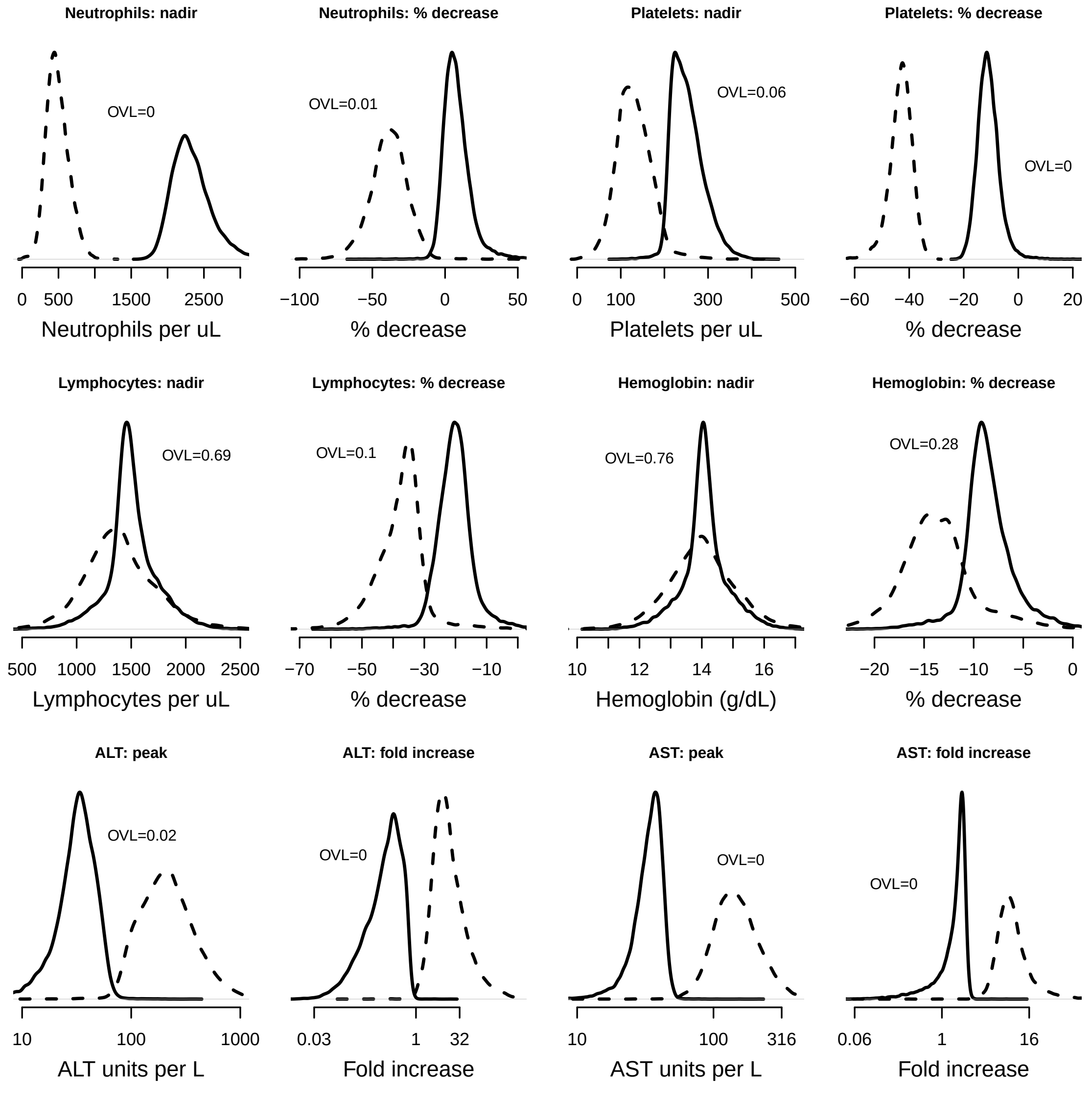

Figure 3: **Posterior distributions over the $E_{\min}$ (thick lines) and $E_{\max}$ (dashed lines) values for all models and for all pharmacodynamic outcomes of interest.** The overlap coefficients (see equation 2 in main text) rounded to 2 decimal places are given in text for each pair of marginal posterior distributions.

2 Pharmacokinetic modelling

2.1 PK model

The final NONMEM script used to model the pooled PK data was as follows:

```
;;----------------------------------------------------------------------------;;
;; NONMEM model
;;----------------------------------------------------------------------------;;
;; Modeller: James Watson
;; Project name: Fexinidazole - Phase 1,2,3
;; Date: 2018-12-31
;;----------------------------------------------------------------------------;;

$INPUT
ID TIME AMT DV CMT EVID MDV RATE=DROP DOSA FOOD BW CHAGAS HAT ETH
$DATA AugmentedData_pooled.csv
IGNORE = @
$SUBROUTINE
  ADVAN5 TRANS1
$MODEL
  COMP  = (1) ; Gut
  COMP  = (2) ; M2
  COMP  = (3) ; transition cpt 1
  COMP  = (4) ; transition cpt 2
$PK
  BWMED    = 50;
  TVMT     = THETA(1);
  MT       = TVMT*EXP(ETA(1));
  TVCL     = THETA(2);
  CL       = TVCL * EXP(ETA(2)) * (BW/BWMED)**(0.75);
  TVV1     = THETA(3);
  V1       = TVV1 * EXP(ETA(3)) * (BW/BWMED)**1;
  TVF1     = THETA(4);
  TVHAT    = THETA(5);
  TVCHAGAS = THETA(6);
  F1       = TVF1 * (1 + TVHAT * HAT + TVCHAGAS * CHAGAS) * EXP(ETA(4))
  S2       = V1
  NN       = 2
  KTR      = (NN+1)/MT
  K13      = KTR
  K34      = KTR
  K42      = KTR
  K20      = CL/V1
$ERROR
  IF(CMT.EQ.2) IPRED = A(2)/S2
  IF(CMT.EQ.2) W     = SQRT(SIGMA(1,1))
  IF(CMT.EQ.2) IPRED = LOG(IPRED)
  IF(CMT.EQ.2) Y     = IPRED+W*ERR(1)

  IRES  = DV-IPRED
  IWRES = IRES/W
$THETA
 (0, 5.65)             ; 1.TVMT
 (0, 6.35)             ; 2.TVCL
 (0, 238)              ; 3.TVV1
 (1 FIX)               ; 4.TVF1
 (.9)                  ; 5.TVHAT
 (1.5)                 ; 6.TVCHAGAS
```

```
$OMEGA
 .12                    ; 1.IIV_MT
 .014                   ; 2.IIV_CL
 .04                    ; 3.IIV_V1
 .15                    ; 4.IIV_F1
$SIGMA
.3
$EST
MAXEVAL=9999 PRINT=5 POSTHOC METHOD=1 INTER NOABORT SIG=2
$COV
PRINT=E
$TABLE
ID TIME DV AMT CMT MDV EVID CL V1 ETA1 ETA2 ETA3 ETA4 BW IPRED IRES IWRES CWRES CHAGAS
HAT ETH DOSA F1 MT
NOPRINT ONEHEADER FILE=results_OneComp_TwoTransitComp_StudyCoef
```

2.2 Parameter estimates for PK model

Final estimates are given in Table 1.

3 Pharmacodynamic model

Stan model code used to fit the exposure response relationships. This gives the full prior distributions used to fit these PK-PD models.

Stan code for the proportional error model (absolute model):

```
Dose_response_Proportional_Error <- "

functions {
  real sigmoid(real dose, real ed50, real log_slope, real max_effect, real min_effect){
    return min_effect + (max_effect-min_effect)/(1 + exp(exp(log_slope)*(dose-ed50)));
  }
}

data {
  int<lower=0> N;
  real dose[N];
  real<lower=0> response[N];
  real<lower=0> ed50_lambda;
  real max_effect_prior_mu;
  real max_effect_prior_sd;
  real min_effect_prior_mu;
  real min_effect_prior_sd;
  real log_slope_prior_mu;
  real log_slope_prior_sd;
}

parameters {
  real log_slope;
  real<lower=0> ed50;
  real<lower=0> min_effect;
  real<lower=0> max_effect;
  real<lower=0> sigma;
}

model {
  log_slope ~ normal(log_slope_prior_mu, log_slope_prior_sd);
```

```
  ed50 ~ exponential(ed50_lambda);
  max_effect ~ normal(max_effect_prior_mu,max_effect_prior_sd);
  min_effect ~ normal(min_effect_prior_mu,min_effect_prior_sd);
  sigma ~ exponential(1);

  for (j in 1:N){
    real response_mu;
    response_mu = sigmoid(dose[j],ed50,log_slope,max_effect,min_effect);
    log(response[j]) ~ normal(log(response_mu), sigma);
  }
}"
```

Stan code for the additive error model (relative model):

```
Dose_response_Additive_Error <- "

functions {
real sigmoid(real dose, real ed50, real log_slope, real max_effect, real min_effect){
return min_effect + (max_effect-min_effect)/(1 + exp(exp(log_slope)*(dose-ed50)));
}
}

data {
int<lower=0> N;
real dose[N];
real response[N];
real<lower=0> ed50_lambda;
real max_effect_prior_mu;
real max_effect_prior_sd;
real min_effect_prior_mu;
real min_effect_prior_sd;
real log_slope_prior_mu;
real log_slope_prior_sd;
}

parameters {
  real log_slope;
  real min_effect;
  real max_effect;
  real<lower=0> ed50;
  real<lower=0> sigma;
}

model {
log_slope ~ normal(log_slope_prior_mu, log_slope_prior_sd);
ed50 ~ exponential(ed50_lambda);
max_effect ~ normal(max_effect_prior_mu,max_effect_prior_sd);
min_effect ~ normal(min_effect_prior_mu,min_effect_prior_sd);
sigma ~ exponential(1);

  for (j in 1:N){
    real response_mu;
    response_mu = sigmoid(dose[j],ed50,log_slope,max_effect,min_effect);
    response[j] ~ normal(response_mu, sigma);
  }
}
"
```

The model fitting using *rstan* along with prior distribution values:

```
library(rstan)
```

```
setwd('D:/Dropbox/DNDi Fexinidazole/Code/Chagas Analysis/')

dose_response_mod_properror = stan_model(model_code = Dose_response_Proportional_Error)
dose_response_mod_adderror = stan_model(model_code = Dose_response_Additive_Error)

log_slope_prior_mu  = 0
ed50_lambda         = 1/500
log_slope_prior_sd  = 10

rstan_options(auto_write = TRUE)
options(mc.cores = 4)

KK=2 * 10^6
TH = 100
warmup = round(2*KK/3)

system.time((
neutrophil_fit = sampling(dose_response_mod_properror,
                          data=list(
                            N=n_chagas,
                            dose=m2_aucs_chagas,
                            response=summary_data$nadir_neutrophils,
                            ed50_lambda = ed50_lambda,
                            max_effect_prior_mu = mean(summary_data$nadir_neutrophils),
                            max_effect_prior_sd = sd(summary_data$nadir_neutrophils),
                            min_effect_prior_mu = mean(summary_data$nadir_neutrophils),
                            min_effect_prior_sd = sd(summary_data$nadir_neutrophils),
                            log_slope_prior_mu = log_slope_prior_mu,
                            log_slope_prior_sd = log_slope_prior_sd),
                          iter = KK, warmup = warmup,
                          thin = TH)
))
save(neutrophil_fit,file = 'stan_model_output_neutrophils.RData')



neutrophil_relative_fit = sampling(dose_response_mod_adderror,
                                   data=list(
                                     N=n_chagas,
                                     dose=m2_aucs_chagas,
                                     response=100*summary_data$delta_nt/summary_data$baseline_neut,
                                     ed50_lambda = ed50_lambda,
                                     max_effect_prior_mu = mean(100*summary_data$delta_nt/summary_data$baseline_neut),
                                     max_effect_prior_sd = sd(100*summary_data$delta_nt/summary_data$baseline_neut),
                                     min_effect_prior_mu = mean(100*summary_data$delta_nt/summary_data$baseline_neut),
                                     min_effect_prior_sd = sd(100*summary_data$delta_nt/summary_data$baseline_neut),
                                     log_slope_prior_mu = log_slope_prior_mu,
                                     log_slope_prior_sd = log_slope_prior_sd),
                                   iter = KK, warmup = warmup,
                                   thin = TH,control=list(adapt_delta=.9)
                                   )
#traceplot(neutrophil_relative_fit)
save(neutrophil_relative_fit,file = 'stan_model_output_neutrophils_relative.RData')

hemoglobin_fit = sampling(dose_response_mod_properror,
                          data=list(
                            N=length(summary_data$nadir_hb),
                            dose=m2_aucs_chagas,
                            response=summary_data$nadir_hb,
                            ed50_lambda = ed50_lambda,
                            max_effect_prior_mu = mean(summary_data$nadir_hb),
                            max_effect_prior_sd = sd(summary_data$nadir_hb),
                            min_effect_prior_mu = mean(summary_data$nadir_hb),
                            min_effect_prior_sd = sd(summary_data$nadir_hb),
                            log_slope_prior_mu = log_slope_prior_mu,
                            log_slope_prior_sd = log_slope_prior_sd),
                          iter=KK, warmup = warmup,
                          thin = TH)

save(hemoglobin_fit,file = 'stan_model_output_haemoglobin.RData')


hemoglobin_relative_fit = sampling(dose_response_mod_adderror,
                                   data=list(
                                     N=n_chagas,
                                     dose=m2_aucs_chagas,
                                     response=100*summary_data$delta_hb/summary_data$baseline_hb,
                                     ed50_lambda = ed50_lambda,
                                     max_effect_prior_mu = mean(100*summary_data$delta_hb/summary_data$baseline_hb),
                                     max_effect_prior_sd = sd(100*summary_data$delta_hb/summary_data$baseline_hb),
                                     min_effect_prior_mu = mean(100*summary_data$delta_hb/summary_data$baseline_hb),
                                     min_effect_prior_sd = sd(100*summary_data$delta_hb/summary_data$baseline_hb),
                                     log_slope_prior_mu = log_slope_prior_mu,
                                     log_slope_prior_sd = log_slope_prior_sd),
                                   iter = KK, warmup = warmup,
                                   thin = TH
)


save(hemoglobin_relative_fit,file = 'stan_model_output_haemoglobin_relative.RData')


platelet_fit = sampling(dose_response_mod_properror,
                        data=list(
                          N=length(summary_data$nadir_platelets),
                          dose=m2_aucs_chagas,
                          response=summary_data$nadir_platelets,
                          ed50_lambda = ed50_lambda,
                          max_effect_prior_mu = mean(summary_data$nadir_platelets),
                          max_effect_prior_sd = sd(summary_data$nadir_platelets),
                          min_effect_prior_mu = mean(summary_data$nadir_platelets),
                          min_effect_prior_sd = sd(summary_data$nadir_platelets),
                          log_slope_prior_mu = log_slope_prior_mu,
                          log_slope_prior_sd = log_slope_prior_sd),
                        iter=KK, warmup = warmup,
                        thin = TH)
save(platelet_fit,file = 'stan_model_output_platelets.RData')



platelet_relative_fit = sampling(dose_response_mod_adderror,
                                   data=list(
                                     N=n_chagas,
                                     dose=m2_aucs_chagas,
                                     response=100*summary_data$delta_pl/summary_data$baseline_plat,
                                     ed50_lambda = ed50_lambda,
```

```
                                        max_effect_prior_mu = mean(100*summary_data$delta_pl/summary_data$baseline_plat),
                                        max_effect_prior_sd = sd(100*summary_data$delta_pl/summary_data$baseline_plat),
                                        min_effect_prior_mu = mean(100*summary_data$delta_pl/summary_data$baseline_plat),
                                        min_effect_prior_sd = sd(100*summary_data$delta_pl/summary_data$baseline_plat),
                                        log_slope_prior_mu = log_slope_prior_mu,
                                        log_slope_prior_sd = log_slope_prior_sd),
                                      iter = KK, warmup = warmup,
                                      thin = TH
)

save(platelet_relative_fit,file = 'stan_model_output_platelets_relative.RData')


####################################


lymphocyte_fit = sampling(dose_response_mod_properror,
                          data=list(
                            N=length(summary_data$nadir_lymphs),
                            dose=m2_aucs_chagas,
                            response=summary_data$nadir_lymphs,
                            ed50_lambda = ed50_lambda,
                            max_effect_prior_mu = mean(summary_data$nadir_lymphs),
                            max_effect_prior_sd = sd(summary_data$nadir_lymphs),
                            min_effect_prior_mu = mean(summary_data$nadir_lymphs),
                            min_effect_prior_sd = sd(summary_data$nadir_lymphs),
                            log_slope_prior_mu = log_slope_prior_mu,
                            log_slope_prior_sd = log_slope_prior_sd),
                          iter=KK, warmup = warmup,
                          thin = TH, control=list(adapt_delta=.9))
save(lymphocyte_fit,file = 'stan_model_output_lymphocytes.RData')


lympocyte_relative_fit = sampling(dose_response_mod_adderror,
                                 data=list(
                                   N=n_chagas,
                                   dose=m2_aucs_chagas,
                                   response=100*summary_data$delta_ly/summary_data$baseline_lypm,
                                   ed50_lambda = ed50_lambda,
                                   max_effect_prior_mu = mean(100*summary_data$delta_ly/summary_data$baseline_lypm),
                                   max_effect_prior_sd = sd(100*summary_data$delta_ly/summary_data$baseline_lypm),
                                   min_effect_prior_mu = mean(100*summary_data$delta_ly/summary_data$baseline_lypm),
                                   min_effect_prior_sd = sd(100*summary_data$delta_ly/summary_data$baseline_lypm),
                                   log_slope_prior_mu = log_slope_prior_mu,
                                   log_slope_prior_sd = log_slope_prior_sd),
                                 iter = KK, warmup = warmup,
                                 thin = TH
)
save(lympocyte_relative_fit,file = 'stan_model_output_lymphocytes_relative.RData')

################################################
outcomes =log10(liver_summary$peak_ALT)
ind=!is.nan(log10(liver_summary$peak_ALT))
outcomes = outcomes[ind]
aucs = m2_aucs_chagas[ind]
alt_peak_fit = sampling(dose_response_mod_properror,
                        data=list(
                          N = length(outcomes),
                          dose = aucs,
                          response = outcomes,
                          ed50_lambda = ed50_lambda,
                          max_effect_prior_mu = mean(outcomes),
                          max_effect_prior_sd = sd(outcomes),
                          min_effect_prior_mu = mean(outcomes),
                          min_effect_prior_sd = sd(outcomes),
                          log_slope_prior_mu = log_slope_prior_mu,
                          log_slope_prior_sd = log_slope_prior_sd),
                        iter = KK, warmup = warmup,
                        thin = TH
)
save(alt_peak_fit, file='stan_model_output_ALT_peak.RData')
##########################################
outcomes =log2(liver_summary$peak_ALT/liver_summary$baseline_ALT)
ind=!is.nan(log10(liver_summary$peak_ALT))
outcomes = outcomes[ind]
aucs = m2_aucs_chagas[ind]
alt_fold_fit = sampling(dose_response_mod_adderror,
                        data=list(
                          N = length(outcomes),
                          dose = aucs,
                          response = outcomes,
                          ed50_lambda = ed50_lambda,
                          max_effect_prior_mu = mean(outcomes),
                          max_effect_prior_sd = 2*sd(outcomes),
                          min_effect_prior_mu = mean(outcomes),
                          min_effect_prior_sd = 2*sd(outcomes),
                          log_slope_prior_mu = log_slope_prior_mu,
                          log_slope_prior_sd = log_slope_prior_sd),
                        iter = KK, warmup = warmup,
                        thin = TH
)

save(alt_fold_fit, file='stan_model_output_ALT_fold.RData')


##############################################

outcomes =log10(liver_summary$peak_AST)
ind=!is.nan(log10(liver_summary$peak_AST))
outcomes = outcomes[ind]
aucs = m2_aucs_chagas[ind]
ast_peak_fit = sampling(dose_response_mod_properror,
                        data=list(
                          N = length(outcomes),
                          dose = aucs,
                          response = outcomes,
                          ed50_lambda = ed50_lambda,
                          max_effect_prior_mu = mean(outcomes),
                          max_effect_prior_sd = sd(outcomes),
                          min_effect_prior_mu = mean(outcomes),
                          min_effect_prior_sd = sd(outcomes),
                          log_slope_prior_mu = log_slope_prior_mu,
                          log_slope_prior_sd = log_slope_prior_sd),
                        iter = KK, warmup = warmup,
                        thin = TH
)
```

```
save(ast_peak_fit, file='stan_model_output_AST_peak.RData')


outcomes =log2(liver_summary$peak_AST/liver_summary$baseline_AST)
ind=!is.nan(log10(liver_summary$peak_AST))
outcomes = outcomes[ind]
aucs = m2_aucs_chagas[ind]
ast_fold_fit = sampling(dose_response_mod_adderror,
                        data=list(
                          N = length(outcomes),
                          dose = aucs,
                          response = outcomes,
                          ed50_lambda = ed50_lambda,
                          max_effect_prior_mu = mean(outcomes),
                          max_effect_prior_sd = 2*sd(outcomes),
                          min_effect_prior_mu = mean(outcomes),
                          min_effect_prior_sd = 2*sd(outcomes),
                          log_slope_prior_mu = log_slope_prior_mu,
                          log_slope_prior_sd = log_slope_prior_sd),
                        iter = KK, warmup = warmup,
                        thin = TH
)
save(ast_fold_fit, file='stan_model_output_AST_fold.RData')
```

Parameter	Description	Value	RSE	Units
TH 1	1.TVMT	5.65	4.1%	Hours
TH 2	2.TVCL	6.37	5.4%	Liters/Hours/F
TH 3	3.TVV1	239	6%	Liters/F
TH 4	4.TVF1	1*		NA
TH 5	5.TVHAT	0.956	11.7%	NA
TH 6	6.TVCHAGAS	1.55	14.6%	NA
OM 1	1.IIV$_{MT}$	0.129	13.6%	
OM 2	2.IIV$_{CL}$	0.0143	48.3%	
OM 3	3.IIV$_{V1}$	0.0419	22%	
OM 4	4.IIV$_{F1}$	0.152	10.6%	
SI 1		0.391	4.2%	

Table 1: Final parameter estimates from NONMEM PK model. * Fixed value.
